# Editorial: Biological fabrication beyond tissue engineering

**DOI:** 10.3389/fbioe.2024.1435569

**Published:** 2024-06-26

**Authors:** Martyn Dade-Robertson, Pierangelo Gobbo, Thomas E. Gorochowski, Peter Quoc Nguyen, Meng Zhang

**Affiliations:** ^1^ Living Construction Group, Department of Architecture and Built Environment, Northumbria University, Newcastle Upon Tyne, United Kingdom; ^2^ Department of Chemical and Pharmaceutical Sciences, University of Trieste, Trieste, Italy; ^3^ National Interuniversity Consortium of Materials Science and Technology Unit of Trieste, Trieste, Italy; ^4^ School of Biological Sciences, University of Bristol, Bristol, United Kingdom; ^5^ Wyss Institute of Biologically Inspired Engineering, Harvard University, Boston, MA, United States; ^6^ Living Construction Group, Department of Applied Science, Northumbria University, Newcastle Upon Tyne, United Kingdom

**Keywords:** biological biofabrication, engineered living materials, biohybrid materials, biofabrication, functionally graded biomaterials, synthetic biology, bioprogrammable materials, manufacture of biological systems

Biofabrication efforts to date has predominantly focused on tissue engineering applications. However, recognizing the greatly expanding scope and possibilities in biofabrication, we sought contributions that embraced a broader definition of biological fabrication. This approach aligns with Natalio’s (2018) perspective, which combines "… the ability to design bio-inspired molecules (chemistry) with nature’s complexity and ingenious paths (biology) to produce new composite materials with emergent properties while tailoring their end-functionality or functionalities.” We also wished to provide a forum for the growing interest in implementing these biofabrication technologies to fields beyond the typical purview of biomedicine, such as architecture, textiles, and food manufacturing.

We were open to and received a diverse range of contributions. Included in this edition, we explored new methods of bifurcation for cultured meat, such as those proposed by Li et al., who stacked muscle-like layers as scaffolds for expressing proteins to replicate the complex organization of muscle and fat found in animal tissue. Notably, this emerging domain of lab-grown meat fabrication offers a crossover discipline in tissue engineering. Its aim is to develop large-scale industrial processes that bypass the intricate fabrication required for synthesizing organs and tissue cultures for transplantation.

In the process of scaling up, Ho et al. argue for the consideration of cell-free systems, which could be integrated into architectural-scale living skins and membranes. These systems, requiring less maintenance and combining DNA programming with cellular-like 3D printed scaffolds, have demonstrated a new type of biologically interactive material with broad functional and aesthetic applications for buildings.

Another innovative approach, combining 3D printing with biological substrates for building applications–specifically Living Building Materials–is presented by Reinhardt et al. They merge biomineralizing, calcium carbonate-inducing bacteria with alginate hydrogels to print structures lithified by microbes. Integrating a multi-scale approach this project combines digital and biological fabrication, with 3D printing handling macro forms and biology addressing the meso- and nano-scale of fabrication. The project points towards new methods of fabricating building structures, albeit with significant scaling challenges.

In the short to medium term, it is more likely that we will need to consider new types of biohybrid materials, where some structural and organizational aspects are addressed by more traditional fabrication methods. This idea is the focus of Sherry et al., who investigate ‘fiber highways’, demonstrating how bacteria can use fibers within knitted structures as transportation networks. This approach may have applications in, for example, oil spill remediation, as oil-degrading bacteria could be immobilized within these fibrous networks. The paper also highlights the benefits of interdisciplinary collaboration in this emerging research area. While the use of 3D printing can be seen as a natural progression from tissue engineering, where 3D printing of tissue scaffolds is common, the use of knitted structures with microbes leads to emergent functions that may align more closely with, for instance, microbial motility in the rhizosphere of plant roots. We can envision such microbial motility leading to novel functions and fabrication capabilities, especially when combined with biofilm formation.

Lastly, Riedel et al. propose biofabrication not as an end in itself but as a means to produce Engineered Living Material as a therapeutic ‘biofactory’. The biofabrication process here is relatively straightforward, involving the molding of a biocompatible hydrogel. However, the functionally active material, which the authors demonstrate is also chemically controllable, enables a new class of Engineered Living Materials.

The different methodologies highlighted above can only provide an initial overview of the young and emerging research area of biofabrication. In the next future we are expecting a boom of new technologies that will be developed by taking inspiration from the vast diversity of biofabrication methods that can be found in Nature. When will we be able to synthesize materials with the exquisite hierarchical structure of bone? When will we develop the first methods to organize polymers into dissipative vascular networks that consume chemical energy to build matter and self-assemble it into organised structures similar to those found in trees? We believe that the answer to these questions is soon. In order to address these important biofabrication challenges, we need to combine in a synergistic manner the approaches showcased in this themed issue with important advancements in our understanding of cells, tissues, and their fabrication methods.
